# Hybridisation between two cyprinid fishes in a novel habitat: genetics, morphology and life-history traits

**DOI:** 10.1186/1471-2148-10-169

**Published:** 2010-06-08

**Authors:** Brian Hayden, Domitilla Pulcini, Mary Kelly-Quinn, Martin O'Grady, Joe Caffrey, Aisling McGrath, Stefano Mariani

**Affiliations:** 1 UCD School of Biology and Environmental Science, University College Dublin, Belfield, Dublin 4, Ireland; 2 Laboratory of Experimental Ecology and Aquaculture, Department of Biology, University of Rome 'Tor Vergata', Via della Ricerca Scientifica s.n.c, 00133 Rome, Italy; 3 Central Fisheries Board, Swords Business Campus, Swords, Co. Dublin, Ireland

## Abstract

**Background:**

The potential role hybridisation in adaptive radiation and the evolution of new lineages has received much recent attention. Hybridisation between roach (*Rutilus rutilus *L.) and bream (*Abramis brama *L.) is well documented throughout Europe, however hybrids in Ireland occur at an unprecedented frequency, often exceeding that of both parental species. Utilising an integrated approach, which incorporates geometric morphometrics, life history and molecular genetic analyses we identify the levels and processes of hybridisation present, while also determining the direction of hybridisation, through the analysis of mitochondrial DNA.

**Results:**

The presence of F2 hybrids was found to be unlikely from the studied populations, although significant levels of backcrossing, involving both parental taxa was observed in some lakes. Hybridisation represents a viable conduit for introgression of genes between roach and bream. The vast majority of hybrids in all populations studied exhibited bream mitochondrial DNA, indicating that bream are maternal in the majority of crosses.

**Conclusions:**

The success of roach × bream hybrids in Ireland is not due to a successful self reproducing lineage. The potential causes of widespread hybridisation between both species, along with the considerations regarding the role of hybridisation in evolution and conservation, are also discussed.

## Background

Hybridisation is recognised as a potentially powerful mechanism of diversification among vertebrates [1,2]. The introduction of closely related, previously allopatric species [3,4], along with anthropogenic impacts on the environment of sympatric species [5], has lead to the creation of hybrid zones where the opportunity for interbreeding between species is greatly increased. Combined with instances of heterosis, or hybrid vigour, observed in many hybrid zones, this has lead to a proliferation of hybridisation across vertebrate taxa [1,5-10]. Although vertebrate hybrids are rarely as ecologically fit as parental taxa [11], there is an increasing body of evidence supporting the theory that hybridisation can lead to adaptation through the creation of novel genotypes and morphologies [1,5,9,12]. Hybrid taxa with phenotypic traits intermediate between parental species may be able to exploit niches unavailable to the latter, and as such can out-compete them, especially in novel habitats [5,13]. Such a hybrid zone exists between two cyprinid fishes, roach, *Rutilus rutilus *L., and bream, *Abramis brama *L., in many Irish lakes. Hybridisation between members of the cyprinidae is more widespread than in any other group of freshwater fish [14] and extensive hybrid zones exist between closely related cyprinid assemblages across Europe [4,15]. Although locally prevalent hybridisation between more recently diverged taxa such as *A. brama *and *Abrmis bjoerkna *(L.) [16], *Chondrostroma nasus *(L.) and *Parachondrostroma toxostoma *(L.) [17] or *Barbus barbus *(L.) and *Barbus meridionalis *(L.) [18] is facilitated by a comparatively recent divergence [19], roach and bream represent well differentiated genera, thus hybridisation between these species further highlights a readiness to hybridise amongst leuciscinae.

Neither fish is native to Ireland [20,21]: the roach was introduced following the escape of bait fish in one of Ireland's southern rivers in 1889 (the Munster Blackwater) and remained confined to this system until the 1950s and 60s, when it began a rapid colonisation of the country [20]. The exact origins of the bream in Ireland are unknown, while there is no record of their introduction, it is thought that they were first introduced as a food source by monks, arriving from Central Europe during the spread of Christianity. Bream had a patchy and limited distribution prior to an extensive stocking program in the 1950s which established populations around the country [22]. Hybridisation between both taxa was first recorded following the establishment of an invasive roach population in waters containing resident bream stocks [23]. While hybridisation is not uncommon in the native ranges of both fish [24-28], the levels of hybridisation and subsequent success of the roach × bream hybrid in Ireland are unparalleled elsewhere. Kennedy and Fitzmaurice [23] reported 48% of a gill netted sample of fish from Peartree Lough in the Irish midlands to be roach × bream hybrids, outnumbering both parental species. Fahy *et al. *[29] estimated that hybrids constituted between 36% and 71% of the fish fauna of Leixlip Reservoir; although the variation between these estimates highlights a potential bias in their sampling design, the prevalence of hybrids in the reservoir is evident. More recent surveys on the River Shannon, the largest river system in the country, estimate that roach × bream hybrids are the dominant fish in the system, outnumbering both parental species (Central Fisheries Board, internal reports). Conversely, hybrids present in both the UK and mainland Europe, are never reported as an abundant group [30-37].

Although studies of natural populations are scarce, captive breeding studies have demonstrated that F1 hybrids are fertile and progeny have been produced both as F2 (hybrid × hybrid) as well as through back-crossing with either parental species [26,27,31,38,39]. Yet, very little is known about the incidence of natural post-F1 hybrids. The identification of naturally occurring post-F1 hybrids based on phenotypic characteristics alone is not possible [40]. Using anal fin ray counts, Wood and Jordan [26] were unable to differentiate between F1 and F2 roach × bream hybrids. However, Pitts *et al. *[31], using four meristic measurements of the progeny of laboratory-bred crosses, classified back-crossed hybrid morphologies as intermediate between an F1 hybrid and the parent involved in the cross. They were, however, unable to identify any such fish from natural populations. Finally, using a larger dataset and 22 meristic counts, Yakovlev *et al. *[27] observed three morphologies of back-cross progeny: offspring were recorded with a morphology intermediate between parental species and hybrid but also with a fully parental morphology as well as fully F1 hybrid morphology.

Unlike meristic counts, providing information restricted to particular skeletal elements, the development of geometric morphometric techniques allowed quantification and visualization of overall body shape changes [41-43] and their relationships with other variables, improving the ability to conceptually and statistically link ecology with morphology [44,45].

Given the limitations of exclusively phenotype-based methods, their integration with molecular genetic markers represents a significant opportunity to study hybridisation [1,5,10,39]. However, recent studies conducted in Britain have failed to detect evidence of post-F1 roach × bream hybrids in natural populations using genetic techniques [39]. Arguably, the unique Irish populations, characterised by unusually large numbers of roach × bream hybrids, provide the best opportunity to test whether this proliferation simply reflects surprisingly high levels of F1 hybridisation or, the self-propagation of a roach × bream hybrid swarm.

By conducting an interdisciplinary analysis, using geometric morphometrics, two types of genetic markers and life-history traits (age structure and growth rates), we attempt to *a*) investigate the nature of hybridisation in selected environments, *b*) assess the likely ecological processes underlying the observed patterns and *c*) discuss the conservation and evolutionary implications of this phenomenon.

## Results

### Geometric Morphometrics

Geometric morphometrics was carried out on 225 fish in Lough Ramor. Of these, 101, 57 and 67 had been visually identified as roach, bream and roach × bream hybrids respectively. In Ross Lake, 233 fish consisting of 68, 112 and 53 putative roach, bream and roach × bream hybrids were analysed.

**Figure 1:**
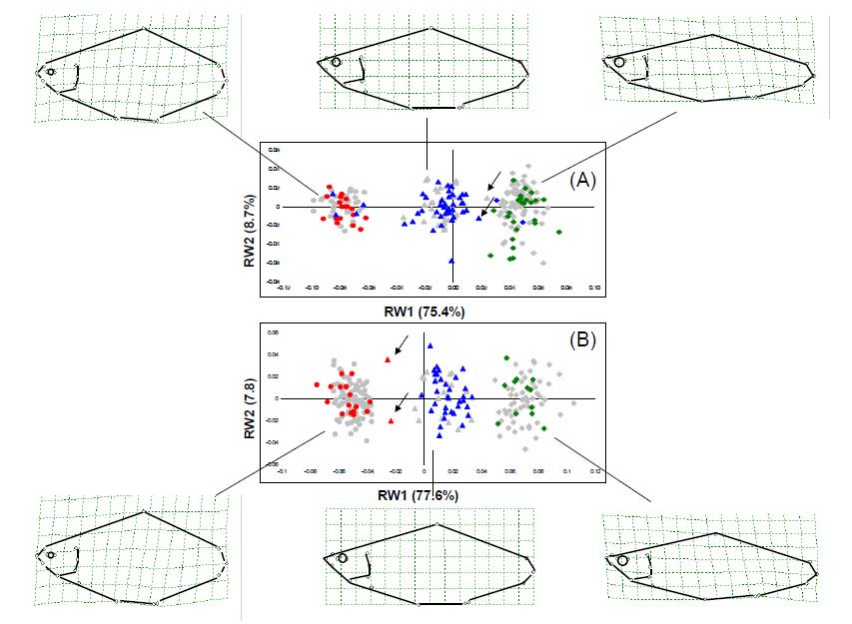
**Cohort size of roach, bream and hybrids in all lakes**. Year class strength of bream, roach and hybrids based on numbers of fish recorded in year class size class.

The first relative warp (RW1) explained the majority of the variation recorded in both Lough Ramor (75.4%) and Ross Lake (77.6), consequently the three taxa could be separated into clearly definable groups along this axis (Fig. 1.). Bream specimens were distributed on the negative portion of RW1, completely separated from roach specimens, located on the positive portion of the axis. Hybrids were located in an intermediate position along RW1. As it is visualized in the splines in Fig. 1, the hybrid body shape can be defined as intermediate between that of the parental species, concerning, above all, the height of the body profile and the caudal peduncle, the length and position of the mouth and the length of the pectoral fin insertion. The output of DFA was highly significant for the two lakes for the comparisons between hybrids and parental species (p < 0.01). In four cases (two fish with a morphology intermediate between roach and hybrid in Lough Ramor and two fish intermediate between bream and hybrids in Ross Lake), DFA was not able to correctly assign the specimens to the a priori identified groups. These individuals could not be confidently assigned to either group based on discriminant function analysis of first relative warp scores (p > 0.05).

Intragroup variation was largely consistent in both lakes although the sample of roach from Lough Ramor, which contained a greater number of juvenile individuals, displayed greater variation in axis 2 that either bream or hybrids (Fig. 1a.). A simple correlation between RW2 and roach size (R = 0.45, p < 0.01) confirmed that variation along RW was mainly accounted for by size.

### Age and Growth Analysis

**Figure 2:**
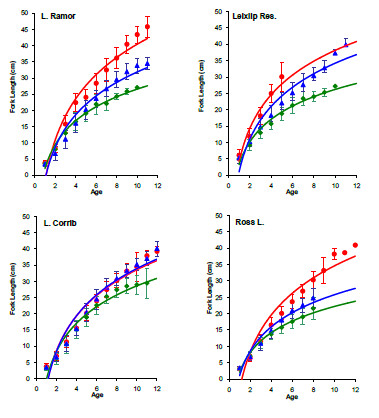
**Location of study sites**. Outline map of Ireland displaying the location of the four lakes sampled for the study. Location of Ireland relative to Northern Europe displayed in inset.

**Figure 3:**
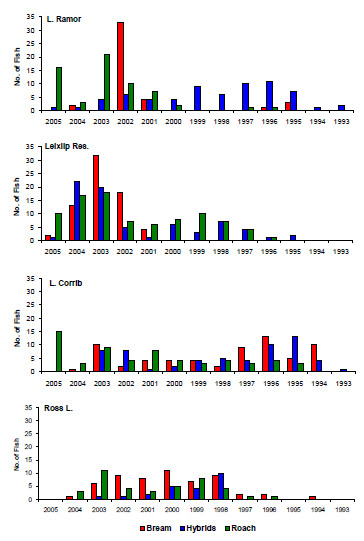
**Landmarks used for morphometric analysis**. Configuration of landmarks (black circles) collected on bream (in the picture), roach and their hybrid. (1) Snout tip; (2) posterior extremity of the premaxillar; (3) nostrils; left (4) and right (5) extremes of the eye; (6) insertion of the operculum on the lateral profile; (7) point of maximum extension of the operculum on the lateral profile; (8) extremity of the operculum; (9) anterior insertion of the dorsal fin; superior (10) and inferior (12) insertion of the caudal fin; (11) posterior body extremity; (13) anterior insertion of the anal fin; (14) anus; (15) insertion of the pelvic fin; superior (16) and inferior (17) insertions of the pectoral fin.

Age and growth analysis was carried out on 697 fish in total, 185, 164, 119 and 229 fish were analysed from Lough Corrib, Lough Ramor, Ross Lake and Leixlip Reservoir respectively (Fig. 2). In the four lakes sampled, growth rates of hybrids and roach were never significantly different. In Ross Lake, Lough Ramor and Leixlip Reservoir growth rates of bream were significantly faster than hybrids and roach, by contrast in Lough Corrib there was no significant difference in the growth rate of either parental species or hybrids. Spearman correlation did not identify a significantly strong negative correlation between year class size of hybrids and either parent species in any lake studied (P > 0.05), as such the hypothesis that hybridisation is more prevalent in years of low parental reproduction cannot be supported (Fig. 3). In the majority of cases no correlation was observed (P > 0.05), indicating that reproductive success for each taxon is independent, in Ross Lake however, a strong positive correlation (P < 0.01) was observed among the three species (Spearman's r_s _= 0.77, 0.88, 0.78, respectively for bream.hybrid, bream.roach and roach.hybrid comparisons). Similarly, a positive correlation was observed between bream and roach in Lough Corrib (r_s _= 0.67, P = 0.02).

### Molecular analysis

A total of 320 fish were analysed, comprising 40 hybrids and 20 of each parental phenotype were selected from each lake. In Lough Corrib, Leixlip Res. and Ross Lake all specimens of bream were homozygous at ITS1, while in Lough Ramor 80% of fish visually identified as bream were homozygous (Table 1.). Similarly in Leixlip Reservoir, Ross Lake and Lough Ramor all roach analysed where homozygous while in Lough Corrib two heterozygous roach were recorded. All putative hybrids analysed were heterozygous at ITS 1 and in the majority of cases amplified cytochrome *b *at 670 bp, indicating that bream were maternal during hybridisation. In Ross L. and L. Ramor all hybrids examined exhibited bream mtDNA, while four hybrids in Leixlip Res. and one hybrid in L. Corrib exhibited roach mtDNA (Table 2).

**Table 1:** Results of genetic analysis of roach, bream and hybrids

Lake	Bream		Roach × bream		Roach	
	**Ho**	**He**	**Ho**	**He**	**Ho**	**He**

L. Ramor	16	4	0	40	18	2
Leixlip Res.	20	0	0	40	20	0
Ross L.	20	0	0	40	20	0
L. Corrib	20	0	0	40	18	2

Genotype of fish screened at ITS1, 40 roach × bream hybrids and 20 individuals from each parent taxa at each lake. Homozygous fish (Ho) amplified PCR product of either parental species, heterozygous fish (He) amplified PCR product of both parental taxa.

**Table 2:** Direction of hybridisation in roach × bream hybrids

	Bream	Roach
L. Ramor	40	0
Leixlip Res.	36	4
Ross L.	40	0
L. Corrib	39	1

Mitochondrial DNA exhibited by roach × bream hybrids, 40 hybrids were screened from each lake. Presence of mitochondrial DNA indicates which species was maternal during hybridisation.

### Post-F1 Hybridisation

Some 154 of the 160 roach × bream hybrids studied were heterozygous with a hybrid appearance (80 of which were also examined through geometric morphometrics). No homozygous fish with hybrid morphology were recorded, while the DFA of morphometric results suggest that most of hybrids are F1 (Fig. 1). In Lough Ramor, four fish with bream morphology and one fish with roach morphology were heterozygous at ITS1, also a second fish with roach morphology, although homozygous for the roach-specific ITS1 band, amplified a cyt *b *band at 670 bp, indicating the presence of bream mtDNA. Two of the fish from Lough Corrib had roach morphology and were heterozygous at ITS1, with one exhibiting roach cyt *b *and the other bearing bream mtDNA (Table 1).

## Discussion

### Nature of hybridisation in Irish lakes

All roach × bream hybrid morphotypes studied in four Irish lakes were heterozygous at the ITS1 nuclear marker, and generally exhibited bream mitochondrial DNA (97%). Allied with geometric morphometric results, whereby the majority of samples were assigned to one of three distinct groups, indicating limited hybrid phenotypic diversity, this indicates that roach × bream hybrids in these habitats are primarily produced as F1 crosses of the parental species, whereby roach males fertilize bream eggs. This finding discounts the view that hybrid swarming is the cause for the extremely high proliferation of roach × bream hybrids. It is worth noting that any heterozygous F2 progeny with a hybrid phenotype, in the sample would not be detectable using this methodology, however to account for the possibility of their existence one would have to assume lethality of F2 individuals intermediate between F1 and parental morphologies, which is not a parsimonious explanation in a scenario where hybrids are so plentiful [46].

Despite such an apparent dominance of first generation crosses, there were varying examples of back-crossed hybrids. Morphometric analysis in Lough Ramor identified two fish with morphologies intermediate between roach and roach × bream hybrids, one of these was heterozygous and the other was not available for genetic analyses. The heterozygote exhibited bream mtDNA and is typical of back-crossed fish recorded in previous laboratory rearing experiments [27,31]. In Ross Lough, both fish exhibiting morphology intermediate between bream and roach × bream hybrids were homozygous for bream ITS1 and also displayed bream cytochrome *b*, indicating that they are likely the progeny of a backcross between a bream and a roach × bream hybrid. However, due to the predominance of bream cytochrome *b *in the hybrid population any attempt to ascertain the direction of the cross using this methodology would be futile.

Two putative back-crossed hybrids with roach morphology were observed in Lough Ramor, though one was heterozygous for ITS1 the other was homozygous for roach ITS1 while both amplified bream cytochrome *b*. Considering that the vast majority of roach × bream hybrids analysed contained bream mitochondrial DNA it is probable that these fish are the offspring of a female roach × bream hybrid and a male roach. Thus while it appears that hybrid swarming is not evident in Lough Ramor, introgression of bream genes into the roach population as a result of backcrossing is a feature of the hybrid zone.

The presence of two homozygous fish with a phenotype intermediate bream and hybrids in Ross Lake either reflects a very unlikely non-recombinant F2/backcross, or perhaps more likely, the action of concerted evolution, such as biased gene conversion which can homogenise nr DNA to that of one parental species after a relatively small number of generations [47]. This trend further highlights the apparent paucity of post F1 hybridisation as a similar return to homozygosity would likely be observed in individuals within the hybrid swarm after a similar number of generations. Although roach are a relatively recent addition to the systems studied, they have been present in most lakes since the early 1980's, providing ample time for at least 15 hybrid generations to occur.

Instances of back-crossing between bream and roach × bream hybrids were also observed in Lough. Ramor. Out of 20 bream morphotypes on which molecular analysis was carried out, four were heterozygous. Predictably, all four fish exhibited bream mitochondrial DNA, which, given the prevalence of this in roach × bream hybrids, makes any prediction of the direction of back-crossing between bream and hybrids impossible. Following Hardy-Weinberg assumptions, an approximately equal number of homozygote bream morphotypes bearing bream mtDNA could arguably be back-crosses between bream and F1 hybrids; therefore the extent of introgressive hybridisation in the lake is probably greater than that which the test can detect directly: eight out of 20 bream morphotypes (40% of the sample) are likely to be back-crosses.

There was also evidence of some back-crossing in Lough Corrib. Two fish visually identified as roach were heterozygous. One of these amplified bream mtDNA while the other exhibited roach mtDNA, showing that natural back-crossing between roach and roach × bream hybrids can occur in either direction.

### Ecological and life-history aspects

Heterosis of the hybrid population was evident in both the growth rate and maximum length attained by the roach × bream in all lakes. The F1 hybrid phenotype, as portrayed by morphometric analysis, was intermediate between both parental phenotypes; consequently it can be assumed that the recorded intermediate mean 'length at age' size of the F1 hybrids is indicative of a healthy population. Similarly, the hybrid exhibited a growth rate which could not be significantly distinguished from that of either parent taxa and as such the intermediate hybrid must be recognised as a successful phenotype in Irish lakes. Similar levels of hybrid vigour where observed in three of the four lakes studied however it should be noted that in Ross Lake the growth characteristics of the F1 hybrid most closely resembled that of roach. While this may be due to a relatively small sample size (n = 23) of hybrids which was dominated by one year class it is also possible that the hybrid was less successful in the mesotrophic conditions found in Ross Lake, the least productive of the four lakes studied.

The overall success of the F1 hybrid is in marked contrast to that of post-F1 generations. The absence of F2 progeny may be explained by poor early development of the offspring. Wood and Jordan [26] provide the only report on the production of F2 progeny. During tank rearing experiments they recorded less than 1% survival of eggs to hatching stage, it should be noted however, that although 70-80% of pure species crosses and up to 55% of backcrosses were successful the authors were unable to produce any F1 hybrid progeny for a roach × bream cross, and thus their low F2 survival rate may be due to factors other than infertility in the F1 generation. Yakovlev *et al. *[27] demonstrated decreasing levels of fitness with successive generations amongst post-F1 hybrids. It is possible that although roach × bream hybrids have the potential to reproduce independently, their progeny are less fit than F1 or back-crossed fish and are quickly selected against in a natural population. A similar selective pressure may be operating on some back-crossed progeny. Previous studies have demonstrated that the progeny of back-crosses tend to have a morphology intermediate between the parent species and the F1 hybrid [26,31]. Morphometric analysis in the present investigation, though confirming the presence of such fish in both Lough Ramor and Ross Lake, did not reveal the existence of significant numbers of intermediate morphologies (Fig. 1) in the 120 hybrids studied, suggesting that such phenotypes may be less successful than either typical F1 hybrids or the parental species.

Bream was the maternal species in the vast majority of hybrids. This is in agreement with Wyatt *et al. *[39] who found that although either parent species could be maternal during hybridisation, most roach × bream hybrids in natural populations exhibit bream mtDNA. Previous authors performed hybridisation in either direction in controlled experiments [27,31,38], and Nzau Matondo *et al. *[38] and recorded no significant difference in the survival to fingerling stage of roach × bream hybrids, related to the direction of hybridisation. The barrier of hybridisation between female roach and male bream is therefore most likely due to ecological factors rather than physiological ones. Male bream employ territorial behaviour during reproduction [22,24,48]. Large male bream occupy and defend territories of optimal reproductive substrate, female bream move through these territories while releasing their eggs. Male roach do not employ a territorial system during reproduction, male and female fish shoal together over spawning areas before reproduction [49]. The opportunity for reproduction between female bream and male roach is likely to be greater than between male bream and female roach: territorial male bream would not leave their territories undefended to follow female roach; also, male bream may not chase male roach away from their territory as they would not be seen as a threat to their reproductive success. Another consideration is the relative abundance of both species. In all the studies mentioned previously, as well as our own recordings, the populations of roach far exceed those of bream. As both species may spawn in the same location at the same time, male bream may find their territories over-run with roach and the opportunity of a female bream egg being fertilized by a roach sperm may far out-weigh the probability of a bream sperm reaching it first. This is particularly evident in light of the results from Lough Ramor, which contains the highest densities of roach, vastly outnumbering bream (J. Caffrey, unpublished data). Dominance of one species relative to the other is likely to increase the levels of introgression and such a skewed population distribution could also help explain the high estimates (40%) of back-crossing between F1 hybrids and bream.

A small number of hybrids in Lough Corrib and Leixlip Reservoir did exhibit roach mitochondrial DNA. Hence, while there may be ecological barriers to the hybridisation between female roach and male bream, these are not insurmountable. The highest incidence of roach mitochondrial DNA in hybrids was recorded in Leixlip Reservoir, whereby the bream population was comprised of predominantly younger fish than in the other three lakes. Poncin *et al. *[48] report young male bream employing a sneak-mating tactic to avoid competition for spawning grounds with larger males, this reproductive strategy would increase the chance for bream sperm to come into contact with roach eggs. Garcia-Vasquez *et al. *[50] recorded hybridisation between salmon (*Salmo salar L.*) and trout (*Salmo trutta *L.) when sneaking male salmon fertilised eggs of female trout and a similar strategy may explain the increased presence of roach mitochondrial DNA in roach × bream hybrids in Leixlip.

### Evolution and Conservation

While this study rejects the hypothesis of a stable and self-sustaining roach × bream hybrid swarm in Irish waters, it does raise interesting questions regarding the possible impact of the roach × bream hybrid on resident fish populations. The population structure of fish communities in Irish lakes changed dramatically following the invasion of roach into Irish waters with numbers of bream declining rapidly [20]. While this was originally thought to be due to competition for food with roach, our results suggest that this is also likely due to competition for spawning areas. In addition, bream populations already under stress from one invasive competitor will be further pressured by the proliferation of a vigorous sympatric hybrid. This hypothesis fits well the scenario observed in Lough Ramor, where the bream population is being genetically "swamped" by the introgression of the roach genome through back-crossing.

Given that hybridisation occurs to a small extent in the native ranges of both parental species, the situation observed in the Irish lakes is certainly peculiar. The key to understand what is occurring perhaps lies in the fact that the studied habitats are relatively novel for both species (especially for roach). As both species spawn on similar substrates, preferring shallow areas rich in vegetation [22,48,51,52], in their native ranges, the opportunity for hybridisation is largely avoided by their staggered spawning periods. Although Diamond [49] recorded bream and roach spawning during the same period in two locations in England, Molls [51] and Lilja *et al. *[53] both recorded roach migrating to spawning grounds earlier than bream. Studies of roach in their native ranges have shown that the onset of spawning occurs usually when water temperatures reach 14-15°C [49,52,54], although spawning has also been recorded at lower temperatures [55,56] and both species will change their spawning time based on environmental factors [54-56]. Kennedy and Fitzmaurice [22] recorded the onset of bream spawning in Ireland at approximately 15°C. It is likely that in the unstable "temperate oceanic" Irish climate the spawning period for roach and bream, when water temperature reaches 14-15°C, may be considerably shorter than in their native ranges, consequently the opportunity for temporal overlap in the spawning of both taxa and resulting hybridisation is increased.

Moreover, Ireland is species-poor relative to mainland Europe [21], hence the roach × bream hybrid is potentially exploiting a niche in Irish waters which elsewhere is occupied by other cyprinid species. Recent eutrophication of Irish waters favours the success of cyprinid taxa over the native salmoniforms in a similar situation to that previously recorded elsewhere [30], and the rapid expansion of roach populations during the 1970's and 80's indicates the wealth of ecological niches available in such waters [20]. The proliferation of roach × bream hybrids in Ireland is a testament to the vigour of the fish: while it's intermediate morphology does not exhibit the traits of transgressive segregation, which is key to the success of other hybrid taxa [5], it nonetheless appears extremely well adapted to conditions in Irish lakes.

Interestingly, the nature and levels of hybridisation in the lakes studied appears to vary significantly in both space and time. For instance, Lough Ramor exhibits significant levels of back-crossing, especially for bream; whereas data on Leixlip Reservoir exhibits a complete absence of any post-F1 reproduction. Levels of hybridisation also vary annually; the hybrid population in Ross Lake is dominated by one year class, whereas in Lough Ramor annual hybrid recruitment appears to be more stable than that of either parental species. Such variations most likely reflect the interplay between colonisation history and environmental fluctuations, which results in a wide variety of hybridisation scenarios that are likely to respond dynamically to climatic changes. Species will change their spawning time depending on environmental factors [54-56] and the effects of these variations in such novel, non-equilibrium habitats are largely unpredictable. Many recent studies allude to the adaptive role of hybridisation in determining patterns of genetic and phenotypic diversity [8-10]. Given the pronounced tendency of cyprinids to hybridise [14], the monitoring of these habitats can provide 'real time' insight into the role of hybridisation and introgression in driving biodiversity trends in response to environmental variation.

## Conclusions

The Irish roach × bream hybrid zone represents a dynamic scenario whereby a vigorous hybrid coexists with both parental species and acts as a conduit for introgression of genes between both species. In the absence of a self sustaining lineage the hybrid population consists primarily of F1 individuals, the offspring of hybridisation between female bream and male roach, though small but significant numbers of back-crossed progeny are also present. The proliferation of hybrids following colonisation of waters by invasive populations of roach highlights potential secondary impacts of species introductions while also shedding light on ecological factors which facilitate widespread hybridisation.

## Methods

### Study Site

**Figure 4:**
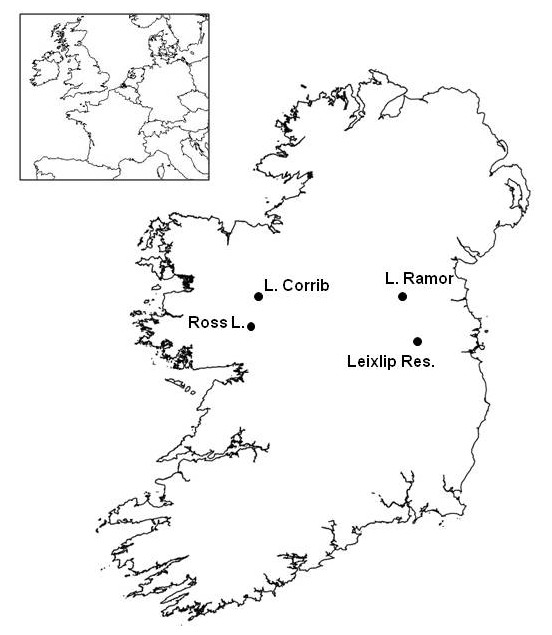
**Geometric morphology and genetics of roach bream and roach × bream hybrids in L. Ramor and Ross L**. Combined relative warp and molecular analyses performed on specimens collected in L. Ramor (A) and Ross L. (B). Splines reflect the shape deformation along RW1. Fish morphometrically assigned as bream, roach and hybrids are represented as circles, diamonds and triangles respectively. Specimens confirmed as homozygous bream or roach by genetic analysis are coloured in red and green while heterozygous hybrids are blue. Putative back-crosses hybrids which were unassigned by the DFA analysis are highlighted by arrows.

Sampling was carried out in four cyprinid dominated lakes in Ireland, selected in order to represent the range of environmental conditions found in Irish freshwater systems (Fig. 4). Leixlip Reservoir (N 53° 21', W 6° 32'), and Lough Ramor (N 53° 49', W 7° 03') are situated on the Liffey and Boyne river catchments respectively. Both water courses are impacted by cultural eutrophication [57,58], are predominantly shallow (<4 m) and contain large but localised macrophyte beds. As macrophyte beds are the preferred spawning substrate for both taxa [22,49,52], the localised nature of these beds could increase the likelihood of bream and roach being forced to spawn in the same areas, thus providing an opportunity for hybridisation. Roach were first recorded in both catchments in 1978 [20], and along with roach × bream hybrids now dominate the fish community of both lakes (Central Fisheries Board, unpublished data; [29]). Lough Corrib (N 53° 27', W 9° 17') and Ross Lake (N 53° 20', W 9° 04') are mesotrophic and contain lower densities of cyprinids than the eutrophic lakes [59,60]. Roach were first recorded in both these lakes in 1980, though it is likely that they were present for some time previously [20]. Lough Corrib (C. 200 km^2^) spans two geological basins (granite and limestone) and contains a wide variety of macrophyte habitats suitable for spawning [61]. In conjunction with the lower cyprinid populations, this is likely to reduce competition between both parental taxa for spawning habitat relative to the eutrophic lakes, possibly resulting in less frequent hybridisation. Ross Lake is a small lake (C. 1.4 km^2^) situated near the Corrib catchment, on limestone bedrock, and is dominated by roach and perch but also contains populations of bream and hybrids [59]. All sampling and analysis were undertaken in conjunction with the Central and Regional Fisheries Boards, Ireland's national fishery management agency, in full compliance with their ethical protocols.

### Age and growth analysis

Fish were initially identified on the basis of their visual appearance and the number of rays in the anal fin. Roach have 8-14 rays in the anal fin and bream have 23-30, while studies of both laboratory reared F1 and natural populations of roach × bream hybrids, which may contain F2 or later generations, report between 16 and 21 anal fin rays [28,31]. A sub sample of fifty individuals, of both parental species and hybrids, were measured (fork length) and a sample of scales (taken between the dorsal fin and the lateral line) was removed for ageing. Scales were subsequently dried and stored in a paper envelope for later analysis. In the laboratory, scales were cleaned with a saline solution, to remove dried mucus and skin cells, and viewed under a Bell & Howell MT633 microfiche reader. Annual checks were recorded as the point where circuli became closely spaced followed by areas of widely spaced circuli. An annual check was only confirmed when associated structures could be viewed around the circumference of the check [62]. At least three scales were read for each fish to confirm the age, and 20% of scales were cross-checked by a second analyst to avoid bias, in cases of irresolvable difference between the measurements of both analysts samples were removed from the study. Age data were used to identify year class structure for each taxon [63,64] and growth rate of each fish was estimated by back calculating length-at-age [65] using the body proportion hypothesis, previously determined as the most accurate method with regard to cyprinids [66]. Length-at-age values were subsequently log transformed to achieve a homoscedastic, normally distributed dataset and ANCOVA was employed to test for significant difference between the growth rates of both species and hybrids. Year class success between hybrids and parental species was tested using Spearman's rank correlation. Strong year class variability has been recorded in both parental taxa [35,64] and it was predicted that hybridisation would be most common in years when one or both parental taxa did not reproduce successfully.

### Biometric data

**Figure 5:**
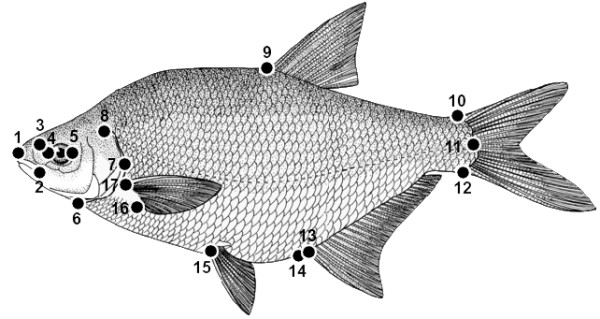
**Growth analysis of roach, bream and hybrids in all lakes**. Growth rate of bream, roach and hybrids derived from mean length-at-age measurements fitted with best fit logarithmic trend lines. Bream, hybrids and roach are displayed as circles, triangles and diamonds respectively, error bars detail standard deviation.

Geometric morphometric analysis was carried out on a subsample of specimens from Lough Ramor and Ross Lake, the same analyses could not be performed on fish from Leixlip Reservoir or Lough Corrib, as the lakes were sampled prior to Lough Ramor and Ross Lake digital images were not available for these fish. A sample of 458 fish, comprised of roach, bream and putative roach × bream hybrids, were analysed (225 from Lough Ramor and 233 from Ross Lake). Each fish was photographed in lateral aspect and digital images were processed as follows: On the right side of each specimen, 17 landmarks (Fig. 5), homologous points bearing information on the geometry of biological forms [41], were digitized using the software TPSDIG [67].

TPSSMALL [68] was used to determine whether the amount of variation in the shape data set was small enough to permit statistical analyses to be performed in the linear tangent space approximate to the non-linear Kendall's shape space. The landmarks were converted to shape coordinates by Procrustes superimposition [69]. This procedure removes information about location and orientation from the raw co-ordinates and standardizes each specimen to unit centroid size, a size-measurement computed as the square root of the summed squared Euclidean distances from each landmark to the specimen's centroid. Residuals from the superimposition were analysed with the thin-plate spline interpolating function [41], producing principal warps. Then, the configurations of the specimens into the principal warps space (the so-called partial warps) were analysed through relative warp analysis (RWA) using TPSrelw [70]. RWA is analogous to a principal component analysis for this type of data [42], with the relative warps being the principal components of shape variables and reflecting the major patterns of shape variation within each species. Significance of shape differences was assessed by a Discriminant Function Analysis (DFA) computed on the matrix of partial warps, using MorphoJ [71] DFA is a supervisionate discriminant analysis that starts with an initially defined grouping of objects that tries to determine to which extent a set of quantitative descriptors can efficiently explain this grouping [72]. In addition, canonical variance analysis procedure in MorphoJ carries out a leave-one-out cross-validation (number of permutation runs = 1000) to assess the reliability of classifications. Specimens which were not unambiguously assigned to either parental group or F1 hybrids after cross-validation were considered to be the progeny of a back-cross between an F1 hybrid and the parental species most closely resembling by the fish.

### Molecular Analysis

Molecular analysis was carried out on a sub-sample of 80 fish, 40 individuals visually identified as hybrids and 20 of each parental species, from each lake. Fin clips were taken from the caudal fin of each fish and stored in 200 proof (>99.5%) molecular grade ethanol. DNA isolation was carried out using a modified chloroform method [73,74]. Following the methodology of Wyatt *et al. *[39], the ITS 1 region of nuclear ribosomal DNA (nrDNA) along with a mitochondrial DNA marker (cytochrome *b*), were used to identify parental species and hybrids.

A multiplex PCR containing two species-specific forward primers, outlined in Wyatt *et al. *[38], and a universal reverse primer was carried out for each marker type. A reaction volume of 25 μl was used in both reactions. The PCR mixture for amplification of the ITS 1 region of nrDNA contained; 10.8 μl H_2_0, 2.5 μl NH_4_^+ ^buffer, 2.5 μl dNTP's, 1 μlMgCL_2_, 1 μl of both species specific forward primers (10 pmol), 1 μl universal reverse primer (10 pmol), 0.2 μl Taq DNA polymerase and 4 μl DNA template. PCR was performed following the thermocycle described by Wyatt *et al. *[39], although optimal results were obtained by increasing the annealing temperature from 55°C to 56°C. PCR products were subsequently subjected to gel electrophoresis on a 1% agarose gel. Successful PCRs amplified products of ~150 bp and ~385 bp for bream and roach respectively, with roach × bream F1 hybrids expected to amplify both products.

Cytochrome *b *amplification took place in a mixture of 9.8 μl H_2_0, 2.5 μl NH_4_^+ ^buffer, 2.5 μl dNTP's, 1 μlMgCL_2_, 1 μl of both species specific forward primers (10 pmol), 1 μl universal reverse primer (10 pmol), 0.2 μl Taq DNA polymerase and 5 μl DNA template. PCR was performed following the thermocycle described by Wyatt *et al. *[39]. PCR product was subjected to electrophoresis as previously mentioned. PCR product amplified at ~670 bp and ~450 bp for bream and roach respectively, with hybrids expected to amplify the band of whichever species was maternal during hybridisation.

### Inference of Post-F1 hybridisation

Combining the results of both methodologies allowed the estimation of the incidence of post-F1 hybridisation and back-crossing. Geometric morphometric analysis was used to visualise the morphology of each specimen while molecular analysis was used to ascertain its genotype and haplotype. Controlled breeding studies have shown most back-crossed hybrids to have morphologies intermediate between an F1 hybrid and the parent involved in the cross [26,27,31]. Such fish are readily identified using geometric morphometric techniques such as those employed in this study.

In order to identify post-F1 hybrids information from biparentally and maternally inherited molecular markers was used. Mitochondrial DNA was used to discern which species was maternal during hybridisation, while the nuclear marker was used to assign the genotype of the fish [75]. Following classic Hardy-Weinberg assumptions homozygous fish with typically hybrid morphology would be considered F2 [76,77], however as ITS1 is a multicopy region, it is unlikely, due to recombination of both parental genes in the F1 progeny, that pure homozygous hybrids would exists in an F2 individual. Therefore morphometric analysis was utilised to infer the prevalence of post F1 individuals within the hybrid population. Previous studies have shown that recombination in post-F1 hybrids leads to greater variance in meristic and morphological traits in F2 and subsequent populations than F1's [1,5]. Ultimately resulting in a blending of hybrid and typical parental phenotypes, generating a near-continuous distribution with poorly distinguished groupings and low classification power using canonical (discriminant) analysis [78]. The presence of such a continuous morphological distribution between roach and bream would indicate that successful reproduction between hybrids is widespread in the systems examined.

## Authors' contributions

BH carried out molecular analysis, contributed to morphometric and growth analysis and drafted the manuscript. DP performed morphometric analysis, contributed to statistical analysis and drafted relevant sections of the manuscript. AMcG carried out fish ageing analysis. MO'G, JC and MKQ conceived the study, contributed in its design, and helped to draft the manuscript. SM orchestrated both the geometric morphometric and genetic aspects of the study, coordinated laboratory based aspects of the study and was involved in drafting the manuscript. All authors read and approved the final manuscript.
